# ‘Very hit and miss’: an interpretive phenomenological analysis of ambulance service care for young people experiencing mental health crisis

**DOI:** 10.29045/14784726.2022.06.7.1.43

**Published:** 2022-06-01

**Authors:** Brioney Gee, Helen Nicholls, Sam Rivett, Tim Clarke, Jon Wilson, Larissa Prothero

**Affiliations:** Norfolk and Suffolk NHS Foundation Trust; University of East Anglia ORCID iD: https://orcid.org/0000-0003-0781-7753; Norfolk and Suffolk NHS Foundation Trust; Norfolk and Suffolk NHS Foundation Trust; Norfolk and Suffolk NHS Foundation Trust; University of East Anglia; Norfolk and Suffolk NHS Foundation Trust; University of East Anglia ORCID iD: https://orcid.org/0000-0002-5279-6237; East of England Ambulance Service NHS Trust ORCID iD: https://orcid.org/0000-0002-5440-8429

**Keywords:** ambulances, crisis care, emergency services, mental health, young adults

## Abstract

**Introduction:**

The ambulance service provides vital front line mental healthcare for young people in crisis, but there is a lack of evidence to guide best practice in this area. The lived experiences of service users can offer important insights to guide service development, therefore we carried out a qualitative evaluation of care provided by the ambulance service to young people experiencing a mental health-related emergency.

**Methods:**

Ten participants aged 16–25 years who had used the ambulance service due to a mental health crisis within the past 2 years were interviewed about their experiences and view of the care they received. Interviews were transcribed verbatim and interpretative phenomenological analysis used to explore participants’ individual narratives and identify recurrent themes.

**Results:**

A theme of inconsistent quality of care was evident in all participants’ accounts. Contributing to this superordinate theme were six recurrent themes: positive qualities of individual ambulance clinicians, ambivalence about seeking care, the importance of retaining agency, need for mental health training for ambulance clinicians, need for inter-service collaboration and favourable comparison of the ambulance service to other services.

**Conclusions:**

We identified some examples of good practice, including person-centred care, respect for patient autonomy and attending to physical health needs. However, our findings suggest the quality of ambulance service mental healthcare is not yet sufficiently consistent. In the absence of mandatory high-quality mental health training and evidence-based protocols, the quality of care appears largely dependent on the qualities and experience of individual ambulance clinicians.

## Introduction

Mental health presentations account for a significant and increasing share of the growing demand for emergency healthcare (Andrew et al., 2020; Duncan et al., 2019; Roggenkamp et al., 2018). However, the quality of care for those experiencing a mental health crisis has been recognised as highly variable and lacking parity with physical healthcare (Care Quality Commission, 2015; Crisp et al., 2016; Paton et al., 2016). A scoping review of the international literature on pre-hospital management of mental health-related emergencies (Emond et al., 2019) identified several barriers to the provision of high-quality care, including lack of training, limited treatment options and ineffective relationships with specialist mental health services.

The mental health of young people is of particular concern amid evidence of increasing prevalence of mental health conditions among under 25s (Erskine et al., 2015; Ford, 2020; Griffin et al., 2018; Patel et al., 2007). Mental health services have struggled to keep up with demand, resulting in high levels of unmet need (Children and Young People’s Mental Health and Wellbeing Taskforce, 2015; Sheppard et al., 2018). In the absence of timely support, young people often do not access support until reaching crisis point (Gill et al., 2017; Owens et al., 2016). Therefore, while published data are limited, we would anticipate that a significant proportion of those using the ambulance service in mental health crises would be young people. Given their distinct developmental needs (Patel et al., 2007), there is a need to understand how services can provide effective mental health crisis care to this age group. This is supported by the NHS long-term plan (Department of Health and Social Care, 2019), which included a focus on improving mental health crisis support for young people.

The lived experiences of service users can offer important insights to guide service improvement; however, previous qualitative research on the provision of mental health crisis care has focused primarily on clinicians’ perspectives. Therefore, we undertook a qualitative evaluation of the care provided by the ambulance service to young people in mental health crisis.

## Methods

Semi-structured interviews were transcribed verbatim and analysed according to interpretative phenomenological analysis (IPA) guidelines. IPA (Smith et al., 2009) is an approach ideally suited to nuanced exploration of individual participants’ experiences and perspectives, particularly of topics that are complex and emotionally laden.

### Participants and procedure

Eligible participants were aged 16–25 years and had used the ambulance service within the past 2 years (but not within the past 14 days) due to a mental health crisis. Ten participants were recruited to facilitate idiographic analysis in line with IPA guidelines (Smith et al., 2009). Participants were recruited via a promotional leaflet distributed by NHS and third-sector organisations in the east of England and online via the Norfolk and Suffolk NHS Foundation Trust and East of England Ambulance Service NHS Trust websites and Twitter accounts. The leaflet gave brief details of the purpose of the research and eligibility criteria, and invited young people meeting these criteria to contact the study team by text or email if they would like further information. Those who expressed an interest were sent the participant information sheet and, a minimum of 48 hours after receiving this information, were contacted by a member of the team who answered any questions and, if they wished to go ahead, arranged an appointment to complete the informed consent procedure and interview. Of the 14 individuals who contacted the study team expressing interest, four withdrew their interest or were unable to be contacted.

One interview was conducted face-to-face by authors HN and SR at the young person’s home address, and nine interviews were conducted via telephone by HN or BG. Those involved in interviewing were not employed by the ambulance service to decrease the risk of participants feeling unable to share less positive experiences. Interviewers followed a semi-structured interview schedule (Supplementary 1), designed to elicit a detailed narrative of ambulance service care experiences, how these experiences compared to their expectations and any suggestions for improvement. All interviews were audio-recorded. Mean interview duration was 31 minutes (range 19–51 minutes).

### Data analysis

Interviews were transcribed verbatim by members of the analysis team. Following familiarisation, initial notes were added to the transcript noting linguistic, descriptive and conceptual points of interest. Emergent themes were then developed and refined through regular discussions between the analysis team comprising a post-doctoral research psychologist (BG), an assistant psychologist (HN), a young person with expertise by experience (SR) and a research paramedic (LP). Having completed this process for each transcript individually, recurrent themes across participants were identified and organised into a hierarchical structure.

### Patient and public involvement

To facilitate meaningful co-production, a young person (SR) with lived experience of using the ambulance service in a mental health-related emergency co-wrote the interview schedule, designed the promotional leaflet for the study, was involved in interviewing, transcribing and data analysis and is a co-author of this manuscript.

## Results

### Sample characteristics

Participant characteristics are presented in Table 1.

**Table 1.** Participant characteristics.

Participant pseudonym	Age (years)	Sex	Ethnicity	Most recent contact with ambulance service
**Mia**	19	Female	White British	May 2020
**Jessica**	19	Female	White British	September 2019
**Alex**	17	Prefer not to say	White British	March 2020
**Dylan**	25	Male	White British	March 2020
**Ellen**	24	Female	Chinese	January 2020
**Grace**	24	Female	Black / Black British	January 2019
**Jodie**	20	Female	White British	March 2020
**Freya**	19	Female	White British	July 2020
**Beth**	18	Female	White British	January 2020
**Paige**	18	Female	White British	July 2018

### Summary of recurrent themes

While the analysis was primarily idiographic in focus, there was considerable overlap in the emergent themes. We identified a single superordinate theme of inconsistency in quality of care that was evident in all participants’ accounts. Contributing to this were six recurrent themes (three with sub-themes), all of which were identified in at least seven participants’ accounts (Table 2).

**Table 2.** Recurrent themes.

**Superordinate theme (n** =** 10)**					
‘Very hit and miss’: inconsistent quality of care					
Theme 1 (n = 10)	Theme 2 (n = 9)	Theme 3 (n = 7)	Theme 4 (n = 10)	Theme 5 (n = 10)	Theme 6 (n = 7)
‘I’ve had some great people’: positive qualities of ambulance clinicians	‘Maybe I wanted them to do something about it, but I wasn’t really sure what’: ambivalence about seeking care	‘Let me control the situation’: the importance of retaining agency	‘I think training is really important’: need for mental health training	‘They just need to be more linked up’: need for inter-service collaboration	‘I actually want more services to be like them’: favourable comparison with other services
	**Subtheme:**		**Subtheme:**	**Subtheme:**	
	‘It’s just a mental health problem’: fear of not being taken seriously		‘They have good intentions, but it’s a bit stereotypical’: need for better understanding of mental health	‘I don’t feel like they’re very closely tied in with other services’: lack of joined-up working	
			‘The worst thing for me is … a professional who doesn’t feel quite confident enough’: need for interpersonal skills and confidence ‘They just dressed my wounds and left’: prioritising physical over mental health	‘Just kind’ve keep everyone in the loop’: need for better communication	

The number in parentheses (n) denotes the number of participants for whom this theme was present.

#### Superordinate theme

##### ‘Very hit and miss’: inconsistent quality of care

Those who spoke about more than one episode of care (n = 9) described considerable variability in the quality of care on each occasion. For instance, when asked how her most recent experience compared to other occasions on which she had come into contact with the ambulance service, one participant responded:

*I’d say it’s a 50/50 split, I’d say that was bang on average, I’d say about half of my other experiences were a lot more positive, but then the other half were like worse.* (‘Mia’, 19)

Participants also spoke about variability in the skill levels of ambulance clinicians. Some were perceived as highly skilled in supporting young people experiencing a mental health crisis, while others were seen to lack the necessary knowledge, confidence and motivation. For instance, one participant commented:

*there was definitely some people that are quite, seem like they had good experience in mental health and then there was maybe a few examples where they clearly didn’t understand it.* (‘Dylan’, 25)

Another remarked:

*sometimes it’s felt like, it’s just a job, we’ll take her ’cause it’s just a job, whereas other times it’s felt more like, genuinely we care.* (‘Beth’, 18)

There was also a notable contrast between the largely positive appraisal of the physical healthcare provided and often more negative views of mental healthcare. One participant expressed that she felt the ambulance crew who attended would have ‘just turned around and left’ if it hadn’t been for her physical health. She went on to suggest that she would be reluctant to contact the ambulance service for a mental health-related emergency in future:

*if obviously I had a physical issue then perfect … but if I was in that kind’ve situation again and I was attempting to take my life then, I think I would rather just stay at home and sleep it off.* (‘Grace’, 24)

#### Theme 1

##### ‘I’ve had some great people’: positive qualities of ambulance clinicians

All participants commented positively on the personal qualities or interpersonal skills of some ambulance clinicians, using adjectives such as ‘kind’, ‘caring’, ‘supportive’, ‘nice’, ‘understanding’, ‘friendly’ and ‘respectful’. Several participants mentioned that they particularly valued individual clinicians who ‘took the extra time’ to speak with them and try to understand their experience.

Authenticity was valued by participants. Taking the time to do things such as introduce themselves, explain what they were doing and why and share their own experiences was interpreted as indicative of the clinicians’ genuine concern for their well-being. For one participant, the authentic concern of a paramedic helped to mitigate, to some extent, the negative impact of a comment she found insensitive:

*I remember one thing that was quite poignant from my experience of the ambulance service where there was a paramedic who kept telling me, it’s a silly choice to overdose. I mean [laugh] to be honest, he was, he did care so it was better than not caring but it was also quite a strange thing to say.* (‘Ellen’, 24)

Another participant mentioned the importance of the relationship between crew members:

*I think how they get on kind’ve helps as well … sometimes like they get along really well, and bubbly and they’re happy and it just kind’ve makes you feel a bit better.* (‘Freya’, 19)

#### Theme 2

##### ‘Maybe I wanted them to do something about it, but I wasn’t really sure what’: ambivalence about seeking care

Most participants expressed a degree of ambivalence about receiving emergency care, a third party often having called the ambulance service on their behalf. When asked about what they had hoped would happen when the ambulance arrived, one participant replied:

*well I was kinda hoping that I wouldn’t be there for them to help me.* (‘Alex’, 17)

The expectation of conveyance to hospital was a source of ambivalence for some participants:

*when the ambulance did get called, I was really upset I was like I don’t want to go to hospital, but I knew that I needed to.* (‘Jessica’, 19)

Reasons for not wanting to be conveyed to hospital were varied. For some, this was linked to negative past experiences. For ‘Jessica’, her ambivalence seemed to relate to a fear of losing agency (‘I don’t want anyone to know; I just want to deal with myself’).

##### Subtheme: ‘it’s just a mental health problem’: fear of not being taken seriously

A fear of not being taken seriously, or being viewed as an ‘attention seeker’, contributed to ambivalence about seeking care for many participants. For some, this fear seemed to be linked to the perception that the ambulance service is only for physical health emergencies:

*I wouldn’t wanna call if I hadn’t hurt myself or like tried to kill myself, ’cause then I’d feel like an attention seeker.* (‘Mia’, 19)

Paradoxically, some reported they would not seek care from the ambulance service unless they were ‘bad enough’, but that at this stage they would struggle to engage with the support offered:

*by the time I’d want to hurt myself or kill myself then I wouldn’t want their support anyway.* (‘Mia’, 19)

Another participant commented:

*if it wasn’t bad enough that someone else would have to call them, then I wouldn’t really wanna use their time.* (‘Paige’, 18)

#### Theme 3

##### ‘Let me control the situation’: the importance of retaining agency

The importance of preserving choice and autonomy in a potentially disempowering situation was evident in most participants’ accounts. For some, this theme was associated with a lack of agency:

*they weren’t asking me if they could like what’s it, do the, the necessary obs [observations], they just pulled me about.* (‘Freya’, 19)

For one participant, a perceived lack of agency when being attended to by a male paramedic was particularly distressing due to prior negative experiences involving men:

*I like struggle with men and stuff and I was left with this random man … he was just pulling the covers away from me and stuff and I just freaked out even more.* (Jessica, 19)

Other participants recounted positive experiences of having their agency preserved:

*one time I asked the ambulance to stop and they did, they sort’ve, I remember that quite well and that just made me feel like I had more control in a situation where I didn’t have a lot.* (‘Beth’, 18)

Such instances were experienced as particularly meaningful by individuals who had previous experience of disempowering situations, such as restrictive interventions while in in-patient care.

#### Theme 4

##### ‘I think training is really important’: need for mental health training

Increased training was recommended to counter inconsistency in ambulance clinicians’ level of understanding, skills and confidence.

##### Subtheme: ‘they have good intentions, but it’s a bit stereotypical’: need for better understanding of mental health

Participants recounted multiple episodes of apparently well-intentioned clinicians behaving in ways, or saying things, they found unhelpful or insensitive. This often appeared to be due to misconceptions about mental health. For instance, a participant who had self-harmed was told ‘you’re too pretty to hurt yourself’, which she saw as reflecting a misinformed stereotype about why young women hurt themselves.

Another participant who recounted being told overdosing was a ‘silly choice’ commented:

*it would be good if people could be trained to understand that well, it’s not really like much of a choice, when people feel really overwhelmed.* (‘Ellen’, 24)

She emphasised the importance of understanding the contextual factors that might lead to self-harm.

##### Subtheme: ‘the worst thing for me is … a professional who doesn’t feel quite confident enough’: need for interpersonal skills and confidence

Participants spoke about some ambulance clinicians demonstrating limited skills in supporting a young person experiencing a mental health crisis:

*[he was] really like hectic and frantic and it just made things like a lot scarier than it should’ve been.* (‘Jessica’, 19)

The importance of strong interpersonal skills was also emphasised:

*obviously when dealing with mental health it’s quite important to get them all nuanced in personal skills.* (‘Dylan’, 25)

Another participant suggested that ambulance clinicians should complete placements within mental health services.

##### Subtheme: ‘they just dressed my wounds and left’: prioritising physical over mental health

Some participants felt that their mental health was overlooked in favour of addressing their physical health needs:

*they don’t acknowledge the mental health aspect, just try and do the physical, ’cause that’s what they’re comfortable with.* (‘Mia’, 19)

Training was suggested to address this delivery gap between physical and mental healthcare. However, this experience was not universal:

*I think one thing that stood out for me was although they did, the paramedics dealt really efficiently with sort’ve like the physical problem, my mental health was never dismissed or sort’ve like forgotten.* (‘Beth’, 18)

Where participants did report feeling that their mental health was given equal attention to their physical health, they interpreted this as individual clinicians going above and beyond as opposed to something to be expected.

#### Theme 5

##### ‘They just need to be more linked up’: need for inter-service collaboration

##### Subtheme: ‘I don’t feel like they’re very closely tied in with other services’: lack of joined-up working

Participants’ accounts illustrate how a lack of joint working between the ambulance service and partner organisations can result in poor patient experience:

*there was a lot of miscommunication between the ambulance crew and the police … I should’ve been admitted to the hospital, um and the police couldn’t go anywhere because of my risk and everything, and the hospital didn’t want to take me because they know my history, so they were like no we don’t want her … so it was just 2 or 3 hours of going backwards and forwards.* (‘Freya’, 19)

The use of the phrase ‘we don’t want her’ suggests that the meaning of this incident for Freya was that she was being personally rejected, as opposed to services working together to determine the service best placed to meet her needs.

Participants also suggested that, for those not conveyed to hospital, the ambulance service should refer young people directly to relevant mental health services to limit delays in access to specialist care.

##### Subtheme: ‘just kind’ve keep everyone in the loop’: need for better communication

Participants emphasised the importance of good communication to ensure effective care transitions and access to ongoing support. Several spoke about the importance of ensuring a ‘proper handover’ to hospital staff and good-quality medical notes and written communication:

*even when we got to the hospital they were talking to me obviously while they like booked me in, and they explained to me that it was quite busy and there was going to be a wait, but if I needed, they told me like where to go if I needed to talk to a doctor or if things started to get worse.* (‘Jessica’, 19)

Conversely, another participant conveyed her perception of having been abandoned by the ambulance crew following a poor handover, saying ‘they just dumped me in A&E’.

#### Theme 6

##### ‘I actually want more services to be like them’: favourable comparison with other services

While participants’ accounts revealed areas for improvement, the ambulance service was compared favourably to other services, including acute hospitals, mental health services and the police. Interestingly, the non-specialist training of ambulance clinicians was perceived by some as facilitating a more person-centred approach:

*I actually find it very helpful that paramedics are not psychiatrists … I find that they don’t tend to pathologise you so much … because they don’t have the psychiatric terminology, and I would prefer more services to just treat you as you are, as another person, rather than as a patient with a particular label.* (‘Ellen’, 24)

## Discussion

This qualitative evaluation of young people’s experiences of ambulance service care in a mental health-related emergency suggests considerable inconsistency in care quality. While many participants described some positive experiences, this good practice appeared largely contingent on the personal qualities and experience of individual clinicians. While some ambulance clinicians were described as friendly, caring and respectful, others were experienced as insensitive or dismissive. Participants valued being listened to and offered choices in relation to their care, but some described disempowering experiences.

All participants perceived a need for mental health training for ambulance clinicians. This triangulates findings of a study of ambulance clinicians’ experiences of working with people who have self-harmed (Jenkins, 2017). Participating clinicians acknowledged feeling unprepared to manage the growing rate of mental health-related calls, with ‘I’m not sure what I’m doing’ and ‘I worry about getting it wrong’ emerging as key themes. Training needs identified in this evaluation included understanding of mental health conditions and the factors that might lead to crisis, interpersonal skills and the need for parity of mental and physical healthcare. A recent study of student paramedics (Credland et al., 2020) provides support for a suggestion by one participant that ambulance clinicians would benefit from completing placements within mental health services.

Fear of being viewed as a ‘time-waster’ was central to many participants’ ambivalence about receiving care. Echoing the findings of an Australian study (Ferguson et al., 2019), this fear was underpinned by the belief that mental health crisis is not a legitimate reason to seek ambulance care. This belief was sometimes reinforced by ambulance clinicians addressing only the participants’ physical health needs, reflecting previous research showing that paramedics often view mental health as a secondary consideration outside of their core role (Roberts & Henderson, 2009).

While participants described some instances of effective joint working between the ambulance service and partner organisations, there were also accounts of poor collaboration. The language of rejection and abandonment used by participants to describe these experiences demonstrates the potentially profound impact of such experiences. The need for improved care pathways for those in mental health crisis has recently been highlighted (White, 2021) and is a national policy priority (Department of Health and Social Care, 2019). Pilot programmes (East of England Ambulance Service NHS Trust, 2021; NHS England, n.d.; O’Hara et al., 2016) offer promising models for improved joint working.

Despite identifying areas for improvement, most participants compared the ambulance service favourably to other services. Participants valued the authenticity of ambulance clinicians and suggested that their non-specialist training may facilitate a more person-centred approach. Therefore, while seeking to increase the consistency of the care, it will be important to preserve the existing strengths of ambulance clinicians.

### Limitations

The service evaluation was conducted in a region of the United Kingdom served by a single ambulance trust, which may limit the transferability of our findings. Further, while we advertised the evaluation widely, the sample was self-selecting, and most participants identified as female and White British. Additionally, we initially intended to conduct interviews in person, but due to COVID-19 restrictions most were conducted via telephone. While data from telephone interviews have been found to be largely comparable to face-to-face interviews (Sturges & Hanrahan, 2004), this may have impacted data richness.

## Conclusion

Young people experience the quality of mental healthcare provided by the ambulance service as highly variable. There is a need to improve the consistency of care provided through enhanced training, joined-up services and evidence-based protocols, while preserving the existing strengths of many ambulance clinicians in supporting young people in a person-centred manner.

## Acknowledgements

We would like to thank the young people who participated in the evaluation for so generously sharing their experiences with us. We are also grateful to Bhavna Sidhpara for her editorial and administrative assistance during the preparation and submission of the manuscript.

## Author contributions

BG, TC, JW and LP conceived and designed the evaluation; all authors contributed to recruitment of participants; HN, SR and BG collected the data; BG, HN, SR and LP analysed the data; BG wrote the first draft of the manuscript; all authors contributed to and approved the final version of the manuscript. BG acts as the guarantor for this article.

## Conflict of interest

LP is on the editorial board of the *BPJ*.

## Ethics

The study was reviewed and approved as a service evaluation by Norfolk and Suffolk NHS Foundation Trust (REF: 311219) and the involvement of East of England Ambulance Service NHS Trust was approved by the Trust’s medical director. Written informed consent was obtained from all participants.

## Funding

This project was funded by a capacity building grant from the Applied Research Collaboration (ARC) East of England, part of the National Institute for Health Research (NIHR). The views expressed in the manuscript are those of the authors and not necessarily those of the NIHR or the Department of Health and Social Care.
